# Cultural Mechanisms of Leprosy-Related Stigma: A Gendered Analysis Using the What Matters Most Framework in Far-Western Nepal

**DOI:** 10.1177/10497323251318604

**Published:** 2025-03-27

**Authors:** Marlies J. Visser, Eliza A. KC, Yoslien Sopamena, Pirt B. Bist, Sita Bist, Madhusudan Subedi, Nand Lal Banstola, Sarju S. Rai, Lawrence H. Yang, Dadun, Ruth M. H. Peters

**Affiliations:** 1 Athena Institute, Faculty of Science, 1190 Vrije Universiteit, Amsterdam, The Netherlands; 2 1229 Amsterdam Public Health Research Institute, Amsterdam, The Netherlands; 3 Faculty of Public Health, 64733 Universitas Indonesia, Jakarta, Indonesia; 4 Faculty of Humanities and Social Sciences, 523821 Far Western University, Mahendranagar, Nepal; 5 School of Public Health, 415559 Patan Academy of Health Sciences, Kathmandu, Nepal; 6 NLR Nepal, Kathmandu, Nepal; 7 School of Global Public Health, 5894 New York University, New York City, NY, USA; 8 School of Nursing, Duke University, Durham, NC, USA

**Keywords:** leprosy, stigma, culture, gender, what matters most

## Abstract

Leprosy-related stigma in Nepal adversely affects socioeconomic and health outcomes. The cultural shaping of stigma is often overlooked in stigma (reduction) research. Therefore, this study aimed to (i) identify cultural capabilities in daily life that “matter most” for men and women; (ii) extend and corroborate perspectives and experiences of leprosy-related stigma; and (iii) explore how “what matters most” (WMM) intensifies or protects against leprosy-related stigma in Far-Western Nepal. We performed a directed content analysis of 38 interviews and 8 focus group discussions with a total of 80 men and women affected by leprosy, family members, and healthcare workers—directed by the WMM framework. WMM included key personal and family capabilities, centered around personal and family honor and prestige (*ijjat*). Stigma was rooted in cultural beliefs of leprosy as *karma* or *divine punishment* leading to *apahelana* (disrespect or disregard) and social exclusion, indicating a loss of personhood. This hinders attainment of WMM in both family and community settings which can negatively affect self-confidence and (mental) health, exacerbating stigma. The study supports the applicability of WMM to leprosy and found that family support and involvement alongside treatment could mitigate some powerful aspects of stigma attached to leprosy. Given its dual role found in this study, family support should be leveraged for stigma reduction efforts enabling families to facilitate WMM for persons affected by leprosy. This study advances the work on WMM and stigma by exploring an infectious condition historically associated with deeply rooted misconceptions, fear, and exclusionary practices.

Leprosy is a neglected tropical disease (NTD) caused by *mycobacterium leprae* transmitted via droplets from the nose and mouth and it mostly affects the skin and peripheral nerves (Walker & Lockwood, 2006). While leprosy is not commonly associated with tropical regions, it is understood as an NTD given its intricate link with poverty and densely populated communities (Oktaria et al., 2018; Pescarini et al., 2018). Leprosy is curable and is commonly treated with multidrug therapy. However, if not treated in time it may cause irreversible damage to the skin and nerves, which has been called “preventable disability” (van Brakel et al., 2012). In addition, leprosy reactions can occur before, during, or after multidrug therapy, indicating immune inflammatory responses such as acute inflammation in skin lesions and/or nerves, skin nodules, and fever (Putri et al., 2022).

Leprosy remains stigmatized, manifesting as negative attitudes and behaviors toward persons or groups affected by leprosy and/or those with visible deformities or disabilities, often leading to restricted social participation (Adhikari, Shrestha, et al., 2013; Rahman et al., 2022; Stevelink et al., 2011; van Brakel et al., 2019). Stigma refers to a process of negative stereotyping leading to negative attitudes and behaviors (i.e., prejudice and discrimination), perpetuated by power dynamics and structural discrimination (Link & Phelan, 2001). Stigma can be both public and internalized. Public stigma encompasses enacted and anticipated stigma. Enacted stigma refers to the endorsement of negative stereotypes resulting in prejudice and discrimination by others, while anticipated stigma involves the fear and anticipation of such treatment. Self-stigma involves the internalization of public stigma along with negative feelings or impact on the self (Scambler, 2009).

Leprosy-related stigma is driven by cultural and religious beliefs, fear of transmission, and visible skin reactions or impairments (Sermrittirong & Van Brakel, 2014). Stigma is a key factor compounding the social, economic, and psychological burden of leprosy in Nepal, which is influenced by gender (van Dorst et al., 2020; van Netten et al., 2021). Nepal is among the 12 countries reporting between 1000 and 10,000 new leprosy cases in 2022, with 2285 newly detected leprosy cases (WHO, 2023). In this context, leprosy is often considered impure and a divine punishment for past sins, reinforcing the idea of karmic justice (Engelbrektsson & Subedi, 2018). Furthermore, a study from Western Nepal reported that illiteracy, perceived economical inadequacy, a change of occupation, and a lack of knowledge of leprosy were all associated with increased perceived leprosy-related stigma (Adhikari, Shrestha, et al., 2013, Adhikari et al., 2014). Stigma can significantly impact multiple facets of life among leprosy-affected individuals in Nepal, such as social engagement, employment opportunities, marriage, food sharing, physical well-being, and mental health (Marahatta et al., 2018; Pierneef et al., 2022; van Netten et al., 2021; van’t Noordende et al., 2016). Given the fear and risk of discrimination within communities, people often choose to conceal leprosy to maintain their social status, even from their own family members (Adhikari, Kaehler, et al., 2013; Marahatta et al., 2018; Peters et al., 2018). However, concealment is a double-edged sword and can impose additional psychological burdens on individuals and negatively impact mental health and post-exposure prophylaxis strategies, for example (Pachankis, 2007; van Brakel et al., 2019).

Stigma is multifaceted and shaped by complex sociocultural factors. Underlying beliefs, experiences, and outcomes of stigma can vary by cultural context and may differ between individuals and groups (Stangl et al., 2019). Leprosy-related stigma is influenced by socioeconomic interactions, shaped by individual characteristics such as gender and ethnicity and their relationship with privilege and oppression in a certain context (Rai et al., 2020; Stangl et al., 2019). In Nepal, cultural and socioeconomic interactions are deeply intertwined with Hinduism and social understandings of Hindu society (Pradhan, 2011). These interactions are embedded in hierarchical caste structures and patriarchal norms, resulting in caste and gender-based discrimination (Nepali et al., 2018). For example, Adhikari, Shrestha, et al. (2013) found that leprosy-related stigma was highest among Brahmins (deemed “highest caste”) and Dalits (deemed “lowest caste”). Additionally, studies reported a higher risk of late reporting for treatment, higher levels of depression and poorer mental health, and an increased vulnerability to violence and abuse among women living with leprosy (van Dorst et al., 2020; van’t Noordende et al., 2016; Varkevisser et al., 2009).

While the differences in leprosy-related experiences and outcomes in Nepal are evident, the mechanisms by which leprosy-related stigma toward men and women is culturally shaped remain underexplored. This gap in knowledge considering stigma as a deeply social and cultural process hampers the development of effective stigma reducing interventions adjusted to local contexts and responsive to the intricacies of individuals’ lived experiences (Gurung et al., 2022; Yang et al., 2007, 2014). Yang and colleagues posit that a way through which to understand how stigma exerts its core effects within a specific cultural context is by considering stigma as embedded in moral life. As such, and drawing from prior foundational work (Kleinman, 1999), stigma is theorized to relate to a loss of moral experience or that what is most at stake (Yang et al., 2007, 2014). Moral experience is defined as “that register of everyday life and practical engagement that defines what matters most for ordinary men and women” (Yang et al., 2007, p. 1528). “What matters most” (WMM) thus signifies meaningful community participation in a local cultural context such as a neighborhood, a group, a workplace, or a village, and the WMM framework supports the identification of core cultural capabilities that matter most, illuminating the experience of stigma in a specific cultural context (Yang et al., 2007, 2014; Yang, Poku, et al., 2021).

The framework is relevant to all individuals within a local community, extending our scope beyond just those who stigmatize and are stigmatized, and inviting the inclusion from perspectives of different stakeholders to better understand stigma. Moreover, engagements under WMM can both intensify and protect against stigma, in that stigma is felt most powerfully when it undermines WMM, while achieving key cultural capabilities under WMM can protect against stigma (Misra et al., 2021; Yang et al., 2014; Yang, Poku, et al., 2021). The framework has previously been adopted in the United States and Botswana, among other contexts such as Chile (Becker et al., 2023), with studies indicating that financial responsibility or motherhood can be key moral engagements influencing stigma (Misra et al., 2021; Yang et al., 2014; Yang, Poku, et al., 2021). Finally, WMM has been used to operationalize culturally salient quantitative scales for stigma (Yang, Ho-Foster, et al., 2021) as well as guide stigma interventions for women in Botswana (Yang et al., 2022).

To advance the understanding of the mechanisms through which leprosy-related stigma is culturally shaped toward men and women, the aim of our study is threefold: (1) to identify the cultural capabilities that “matters most” for men and women; (2) to corroborate and extend perspectives and experiences of leprosy-related stigma; and (3) to explore how WMM intensifies or protects against leprosy-related stigma, by exploring the perspectives and experiences of people affected by leprosy, their family members, and health workers residing in two districts in Far-Western Nepal. To the best of our knowledge, no study has previously used WMM to inform primary qualitative data collection on leprosy-related stigma in Nepal. Additionally, this study builds on previous work to illuminate if and how cultural processes via WMM can intensify or mitigate stigma related to leprosy (Ebenso et al., 2019). Leprosy is an infectious condition transmitted through prolonged close contact and is historically linked to divinely instituted exclusion to leprosaria and leprosy colonies. This association continues to have an impact today through deeply rooted misconceptions, fear of transmission, and social exclusion (Adhikari, Kaehler, et al., 2013; van Brakel et al., 2019). This study therefore contributes to the stigma and WMM literature by exploring whether cultural processes via WMM can still play a role in shaping stigma related to leprosy.

## Methods

This study is part of a larger three country mixed-methods study that aims to capture and understand culturally salient stigma dynamics by understanding WMM in local contexts in Indonesia, Nigeria, and Nepal, to improve stigma assessment and reduction related to leprosy, lymphatic filariasis (LF), and depressive disorders. The current study focuses on Nepal and leprosy and adopts a qualitative approach in which 80 respondents participated in 38 semi-structured interviews and 8 focus group discussions (FGDs).

### Study Setting

The study is conducted in Sudurpaschim province which refers to the Far-Western region of Nepal. The total population of this province accounted to 2.7 million people in 2021, dispersed across four main regions: a high Himalayan region, Mahabharat region, mid mountains (also referred to as the hilly region), and plain lands of Terai in the south. This study was conducted in Kanchanpur and Kailali districts, two highly populated districts situated in the Terai region. The Brahmin and Chhetri groups account for 60% of the population in this region, followed by Dalits (17%), Janajatis (20%), and Madheshis (1.6%) (Ministry of Economic Affairs and Planning, 2020). The average literacy rate lies at 85.4% for men and 68.2% for women (National Statistics Office, 2023). Hinduism is the main religion (97%) followed by Christianity, Buddhism, and others. Nepal has a pluralist society and is deeply shaped by Hinduism alongside the social organization of Hindu society (Pradhan, 2011), shaping social categories, interactions, norms, and roles tied to WMM.

### Study Population, Sample and Recruitment

Participants were recruited and data was collected between August 2022 and May 2023. Recruitment and data collection was managed by a local research team encompassing a senior and junior field researcher (PB and SB) and a supervisor (MS). Additional support was provided by a local non-governmental organization (NLR Nepal). The field researchers managed recruitment for the interviews and NLR field staff supported and arranged the FGDs from the provincial hospitals in Mahendranagar and Dhangadi. Purposive and snowball sampling were used to identify respondents. Personal information was obtained from service records of primary health clinics, the Early Warning and Reporting government system, and a health personnel data register. In addition, recruited health workers supported the collection of contact information of leprosy-affected people. Inclusion criteria for participants required a leprosy diagnosis within the past 3 years or to be a family member of someone with leprosy included in the study. Participants had to be aged between 18 and 65 years and reside in the study districts. Health professionals were included if they self-reported involvement in leprosy-related healthcare. Exclusion criteria encompassed being outside the specified age range and/or unwillingness to participate in the study. Potential respondents were contacted through telephone communication and interview or FGD dates were planned according to availability. Health workers participated exclusively through individual interviews, whereas persons affected by leprosy and their family members participated either in interviews or FGDs depending on the recruitment approach.

### Data Collection

Before data collection, PB and SB participated in an intensive 3-day online training on leprosy, WMM, and data collection materials. Semi-structured interview and FGD guides were prepared corresponding to the WMM framework and based on questions used in previous studies (Yang, Poku, et al., 2021). The guides, demographic survey, and consent form were first developed in English and translated to Nepali by the field researchers. In case of illiteracy, the study was explained and consent forms were read out to the participants after which consent was provided through a thumb print. The interview and FGD guides included an introduction, questions on “what matters most” to men and women, daily lived experiences relating to stigma, and a closing section for the interview or FGD (see Supplemental Material for items).

We disaggregated data collection by sex, based on prior literature presenting gendered impacts of leprosy and prevailing gender-based discrimination in Nepal (Nepali et al., 2018; van Dorst et al., 2020; van’t Noordende et al., 2016; Varkevisser et al., 2009). As such, the FGDs were sex-specific, meaning that exclusively male or female respondents participated per FGD. However, in each FGD they discussed WMM for both manhood and womanhood. FGDs were conducted with groups composed exclusively of either leprosy-affected individuals or family members and each consisting of 4–6 participants.

The data collection instruments were piloted in August 2022. The guides were revised accordingly after which data collection started that same month. All interviews were organized face to face either in one’s home, at a health facility, or any other place of the respondent’s choice. The FGDs were organized at various locations including NGO and municipality offices and health facilities. After conducting four interviews and one FGD, the field researchers shared their data collection experiences with MS. Feedback was provided regularly. The interviews lasted between 30 and 60 minutes with some extending to 90 minutes, while the FGDs ranged from 60 to 90 minutes. All sessions were audio-recorded and securely stored on a password-protected university drive. Recordings were transcribed verbatim in the local language, translated to Nepali if necessary, and then manually translated to English by the field researchers. To ensure accuracy, a Nepali researcher (EK) cross-checked the English transcripts with the original recordings.

### Data Analysis

The data was analyzed following a primarily deductive and directed qualitative content analysis approach (Hsieh & Shannon, 2005). This enabled the adoption of the WMM theoretical framework to identify core cultural capabilities for men and women and explore how these key engagements relate to leprosy-related stigma in Far-Western Nepal.

First, all coding was performed by MV, EK, and YS using ATLAS.ti 22. The main codes were informed by the coding structures utilized within the larger study on WMM in other settings, as well as derived from previous qualitative research employing WMM (Misra et al., 2021; Sopamena et al., in preparation; Yang, Poku, et al., 2021). The coding tree included five main codes: “stigma experiences,” “what matters most to manhood,” “what matters most to womanhood,” “what matters most and stigma,” and “stigma intersections.” Three authors (MV, EK, and YS) first reviewed 30 transcripts employing an open and inductive coding approach to identify subcodes under each main code. The themes and subcodes were organized into a coding tree including operational definitions (see Supplemental Material). After this, all interviews and FGDs were coded deductively.

Second, two researchers (MV and EK) analyzed all quote extracts grouped under each of the five main codes. FGDs were analyzed at the group level. Key quotes were grouped under the relevant main theme and extracted in an Excel file using multiple sheets to distinguish between quotations from people affected by leprosy, family members, and health workers, as well as between male and female perspectives. Quote extracts were grouped and analyzed separately for men and women using selective coding. Summaries were developed for each key code. This process revealed the key respondent perspectives and experiences of WMM in relation to manhood and womanhood, leprosy-related stigma, and WMM shaping leprosy-related stigma as presented in the Results section.

### Ethical Considerations

The study was approved by the Nepal Health Research Council (reference number 161/2022 P) in July 2022. Informed consent was obtained from all respondents prior to their participation, either through a written signature or a thumbprint. All documents including informed consent forms, demographic documentation, and transcripts were stored on a password-protected university drive.

## Results

This section presents the study sample characteristics and a thematic overview of the perspectives and experiences of men and women affected by leprosy, family members, and health workers on WMM and leprosy-related stigma.

### Respondent Characteristics

Among the total sample, 41 were people affected by leprosy (19 women and 22 men), 19 were family members (8 women and 11 men), and 20 were health workers (10 women and 10 men) (i.e., totaling 37 women and 43 men). Most respondents identified as Hindu (91.2%) and the rest identified as Christian (8.8%). Further, 85% of the health workers belonged to ethnic groups constructed under Brahmin and Chhetri groups, while 26.3% of the family members and 39% of persons affected by leprosy belonged to these groups. Over half of the persons affected by leprosy (56%) and family members (63.1%) belonged to Janajati or Dalit ethnic groups.

The average age among leprosy-affected persons included in the study was 44.9 (standard deviation (SD): 12.4). For health workers and family members, it was 43.4 (7.2) and 46.6 (10.3), respectively. All health workers, family members, and leprosy-affected persons were married, except for one male health worker and five leprosy-affected persons (7.5% of sample unmarried).

All health workers completed a bachelor’s degree. Among the leprosy-affected persons and family members, respectively, 29.2% and 31.6% were either illiterate or had finished primary school and 12.2% and 5.3% finished a bachelor’s degree. Over half of the individuals in both groups (58.5% and 63.2%, respectively) had completed either lower or higher secondary school.

### What Matters Most: Personal and Family Honor and Prestige (Ijjat)

Respondents across all stakeholder groups reported various capabilities for men and women to be considered a “good, proper or complete person” in their community. The analysis revealed that honor and prestige (*ijjat*) relating to the self and the family mattered most for both men and women. While *ijjat* was to a limited extent highlighted by respondents directly reflected in the quote below, our analysis revealed that two subthemes of cultural capabilities were closely tied to personal and family *ijjat*: (1) marriage and family capabilities and (2) personal capabilities, with some specifically tied to WMM to men or women. Table 1 includes the descriptions of these two subthemes as reflected on by key respondents.

Those who have everything, have good food and have ijjat in society. Those who have peace in the family, have children, have land to cultivate. (D29, female family member, FGD)

**Table 1.** Core Themes, Subthemes, and Key Quotes Illustrating Capabilities Under “What Matters Most” Typical Among Women and Men.

WMM		Subthemes	Supporting Quotes
Marriage and family capabilities	General	• Family status • To care for, guide, and educate children	“The one who have a good economic status, a big house, car, motor bike, are considered as complete in our society.” (D24, male health worker, Chhetri, IDI) “Family members and parents must give good education …. they should take care of children, help the young ones to choose the right path….. it is the duty of parents to give good knowledge to children…” (D2, female family member, Madheshi, IDI)
	Women	• To have children • To cater to family well-being and complete household chores (e.g., cooking, feeding children, and taking care of domestic animals) • To balance work and family responsibilities	“Those women who take care of her family and complete all her duties, binds together the entire family, that is called decent women.” (D22, male health worker, Chhetri, IDI) “Getting a job and also fulfilling family duties, [women] must look after their children, balance both house and office work. Women have more responsibility than men.” (D10, female health worker, Chhetri, IDI)
	Men	• To hold financial responsibility and ensure the economic stature of the family	“He must have a well-settled family like children, strong economic status, house, land, he must be able to invest in his children’s education or any other desires …. Economically strong, socially strong. Then he will feel complete, satisfied, …” (D33, male health worker, Brahmin, IDI)
Personal capabilities	General	• To maintain health	“When someone is free from all type of disease and any sorrow, there is happiness in family.” (D1, female family member, Chhetri, IDI)
	Women	• Respectable character, getting along with others, and living in unity • Social participation and acceptance	“She must help others, talk nicely and politely with others.” (D12, women affected by leprosy, FGD) “She must have the quality of getting along with others in society easily, live with unity in society.” (D15, male family member, FGD)
	Men	• Respectable character, to abstain from negative habits (e.g., smoking and alcohol consumption) • Social participation and responsibility	“[A man] has good behavior, [is] polite in nature, doesn’t drink and smoke.” (D19, male family member, Janajati, IDI) “Think about the betterment of his family and society, also live in society with others in a disciplined manner, maintain harmony in society, participate in each and every social activity.” (D20, male health worker, Chhetri, IDI)

When respondents were asked about the consequences of not fulfilling capabilities pertaining to WMM (see Table 1), the majority acknowledged this would have both social and financial consequences for the family. Socially, implications included discrimination, a breakdown of family unity, a lack of respect and support within the family, as well as a lack of proper education of children. These implications were frequently noted in the cases of women not meeting capabilities. Financially, implications manifested through financial strain or shortages within the family. This was a concern commonly cited in relation to men not achieving capabilities to work. Regardless of the unmet responsibility, the majority of respondents recognized their adverse impact on the family:

Earning is the responsibility of men and taking care of the house is the woman’s responsibility. So, she must do her work, otherwise there will be no peace and unity among family members, there will be fights in [the] family. (D15, male family member, FGD)

I: What are the consequences if a man does not complete his duty? R: There will be economic shortage in [the] family, there will be fights, problems in [the] children’s upbringing, their health, food, education … In most cases nowadays a husband and wife get separated. (D11, female health worker, Brahmin, IDI)

### Leprosy-Related Stigma Indicating a Loss of Personhood

Almost all leprosy-affected persons and family members emphasized that leprosy-related stigma persists, noting enacted, anticipated, and self-stigma, facilitated by gossip. Respondents from all stakeholder groups noted that stigma was rooted in both public and internalized cultural beliefs. These beliefs include stereotypes and prejudices about one’s illness, including that leprosy is a consequence of *karma*, *sin from past lives*, or is a *curse from God*:

People talk like God has given it to them, like karma is back. There is … gossip about the one who is affected. The way of seeing the person affected [has] changed. It is considered as the sin of previous birth which has caused it … (D16, male family member, Madheshi, IDI)

I used to think like I’m affected by this disease and if I give it to others then God will curse me. (D13, woman affected by leprosy, FGD)

Respondents across the full sample highlighted that leprosy is associated with public prejudice and discrimination, referred to as *apahelana*, meaning to disrespect and disregard. *Apahelana* conveys a sense of looking down on someone with a lack of respect or contempt, indicating a loss of personhood. To be *apahelit* by society was characterized as being neglected, avoided, and/or isolated. *Apahelana* may lead to exclusion from nearly all aspects of social life which limits participation in WMM. Many respondents across the full sample explicitly stated that once a leprosy diagnosis is known, others tend to *apahelana* the affected individual within community and family contexts:

Usually people apahelana them, dislike them. They say or talk like, “he is affected by the disease, so don’t go near to him, don’t talk to them.” The [people affected by leprosy] feel bad about it, [they] feel left out from society. (D1, female family member, Chhetri, IDI)

My wife gives [me] food separately, [she] does not come near and sleeps in a separate bed. (D30, man affected by leprosy, FGD)

Usually in our village we do all work together, like planting paddy, cutting it. But now they don’t involve me, so I also don’t want to go. Thinking if I go there, they will ask me about my disease. (D12, woman affected by leprosy, FGD)

Stigma further impacts individuals and their families through the anticipation and fear of public stigma, as well as through self-stigma. Self-stigma undermines self-confidence and negatively affects mental health, going as far as suicidal thoughts for some. Respondents noted that both anticipated and self-stigma could severely hamper social participation and thus meaningful participation in WMM:

We think if they know about our disease they will apahelana us, will not come near to us, and not involve us in any function. (D35, man affected by leprosy, FGD)

Like I said earlier in our mind [and] heart, there is only one thing “if they know about us what will they think, what will they say, will they apahelana us.” So, I prefer to not go to such ceremony. (D12, woman affected by leprosy, FGD)

I thought of suicide. I used to think from where this disease came to me. I think about it now also. The doctor made me understand … I used to think of leaving the house. I have some gold on my ear, and I used to think about selling it and to go somewhere. (D44, woman affected by leprosy, other, IDI)

Respondents stated that leprosy is often concealed or that individuals seek treatment at distant health centers to avoid discrimination. In case the person is not able to conceal leprosy due to visible reactions or deformities, stigma is exacerbated. Concealment of leprosy was frequently mentioned by respondents across all stakeholder groups to preserve personhood and participate in WMM:

There is one patient who is having medicine from here only. He can have medicine from his nearby health center, but he does not. … He said that he has doubts or fear that he will be discriminated by villagers if they know about his disease. (D25, male health worker, Chhetri, IDI)

They think it will have effect on the name of family, so they don’t want to be open about their illness. (D15, male family member, FGD)

Some persons affected by leprosy shared they had not experienced stigma. Such perspectives, however, remained the minority of perspectives and experiences shared. Furthermore, several respondents indicated that stigma has been and currently is decreasing due to increased availability of medicines and better community understanding of leprosy transmission and treatment:

No, no one hated [or] ignored me till now. Everyone has treated me nicely … There is no such problem, rather they help me and ask about my condition nicely. Like if I’m taking medicine daily … they treat me like they used to before. (D42, woman affected by leprosy, Rana Tharu, IDI)

### WMM Shaping Stigma

Everyday engagements revolved around the protection of and contribution to personal and family *ijjat*. Leprosy-related stigma was felt most powerfully when it obstructed achieving gendered family capabilities and the attainment of marriage, capabilities related to *ijjat*. This could further exacerbate leprosy-related stigma. On the other hand, family support was observed to contribute to protection against some of the stigma attached to leprosy.

#### WMM Intensifies Stigma

##### Not Fulfilling Gendered Family Capabilities

Being healthy was seen as a necessary precursor to fulfill one’s gendered duties and responsibilities in the family. For women, family members typically expected a woman to be in good health to perform household chores and comply with caretaking roles, emphasized by both male and female respondents. Leprosy-related stigma, especially in case of visible reactions or disability, was observed to affect one’s family and social life challenging the achievement of womanhood:

In our society, … household work depends on women …. Men only do outside work and all household responsibilities are in female hands. So, if women get leprosy and have reactions in her body then she can’t do this work, … if this is the case then she is humiliated from her house, will be apahelana by her family, society. (D23, male health worker, IDI)

Similarly, respondents shared that for men, good health was important to fulfill financial capabilities in the family. Respondents stated that being unhealthy or having impairments could result in a lack of financial stability and dependency on family members for support, challenging the achievement of manhood:

There is difficulty to run the family, look after children’s basic needs, as he is the one who is earning in our family. If he doesn’t go to work then it will be a problem to get food, how we will pay [our] loan. There are many problems. (D3, female family member, Janajati, IDI)

##### Disrupting Marriage Prospects and Family Status

Both persons affected by leprosy and health workers mentioned that marriage prospects could be hampered for affected persons and their family members, even if family members are unaffected. This was linked to an impact on the family status exacerbating leprosy-related stigma:

A few days ago, one woman visited here … she says “everyone knows about this [leprosy] and people are talking bad about our family. My brother wants to get married, but no one wants to marry their daughter to our house.” (D5, female health worker, Tharu, IDI)

Mostly people hide this disease … due to the fear that if anyone knows about it then there will be problem for marrying their children, and it will also affect their family status. (D20, male health worker, Chhetri, IDI)

#### WMM Mitigates Stigma: Family Support to Help People Achieve Ijjat

Family support was raised as a crucial capability to mitigate some powerful aspects of leprosy-related stigma, in combination with undergoing treatment. Examples of family support include support in accessing health services, maintaining respect at home, preserving marriages, and assisting with familial tasks and roles. The support from family members was emphasized to assist leprosy-affected persons in seeking timely care and treatment, which was important to be accepted in the community. Moreover, respondents noted that when persons affected by leprosy have a supportive family, this can motivate and increase their self-confidence which may facilitate participation in WMM:

There is one woman affected by this disease but … she does everything [participates in WMM], she has a supportive family [to facilitate this]. (D9, female health worker, Brahmin, IDI)

Furthermore, respondents across all stakeholder groups shared that family support was needed to ensure concealment of leprosy in social settings. Hence, family support may facilitate family *ijjat* and mitigate some powerful aspects of leprosy-related stigma. However, the persistence of concealment suggests family support may not fully mitigate societal stigma:

Family must support them [affected individuals]. If society’s perspective towards this disease is negative, then [we should] not tell anyone about it …. family members must motivate him, increase their self-confidence. (D18, male family member, IDI)

#### Stigma Intersections: Geographic Location/Ethnicity/Caste and Gender

The data analysis revealed two key intersecting factors of influence on leprosy-related stigma. First, the karmic connotations of leprosy and their implications for stigma were perceived by respondents to vary across ethnic groups, which are socially categorized into castes and related to geographical locations. For example, it was reported that disclosing leprosy would have limited or no impact on the family status in Tharu society when the person is being treated. In contrast, respondents acknowledged that in Pahadi society—associated with the “hilly region” and comprising mostly Brahmin and Chhetri groups considered “higher caste”—leprosy was associated with gossip, discrimination, and exclusion from nearly all aspects of social life by not allowing people with leprosy inside homes or excluding them from daily common chores, such as gathering wood, cutting grass, or farming. This facilitates concealment. This difference was reflected on by health workers:

In Pahadi society they [patients] do treatment and have medicine and they ask us to not to tell others … They say … people apahelana them if they know about [leprosy]. People don’t discriminate like before openly …, people don’t say directly, but they talk. In Rana Tharu, it is easier in their community than in Pahadi. (D33, male health worker, Brahmin, IDI)

Second, gender influences how one is viewed and treated in society driven by patriarchal norms and structures. Consequently, women were perceived to be disproportionately impacted by leprosy. Health workers acknowledged that women are at risk for delayed treatment due to biased affection and care from family members. A woman’s morality and source of disease is often questioned first, which may contribute to losing personal and family *ijjat*:

It is also seen differently if man and women of the same house are affected by leprosy. If the son of that house gets affected then he will be taken good care of, everyone will support him, will treat him immediately. Once the woman gets affected, she will have to listen to many things, like from where she got this disease, her whole family member history will be researched. They say like “she must be affected from her parents’ house only.” (D11, female health worker, Brahmin, IDI)

Health workers reported that in some cases women experience challenges in accessing treatment at the health facility due to the lack of female health workers. Women may be reluctant and feel uncomfortable to reveal their skin to a male doctor:

Mostly here in our hospital there are only male doctors for treatment, so women don’t feel comfortable to do a check-up with a male doctor. That’s why they are left behind in this case. They feel shy to show their wound on [their] body to a male doctor. (D5, female health worker, Tharu, IDI)

## Discussion

In this qualitative study conducted in Far-Western Nepal, we affirmed that public and internalized stigma associated with leprosy persist, leading to far-reaching social, economic, and (mental) health implications for leprosy-affected persons and their families. The study used WMM to explore how leprosy-related stigma is shaped by what it means to be a “proper” and “respected” man or woman. This study advanced the work on WMM and stigma applying WMM to leprosy, an infectious condition transmitted through prolonged social contact deeply rooted in cultural understandings, misconceptions, and exclusionary practices. The study aimed to explore whether cultural processes via WMM can still intensify or mitigate stigma. WMM encompasses personal and family *ijjat*, which in this study was predominantly tied to achieving marriage and family, and personal capabilities. Given their importance in everyday engagements, leprosy-related stigma was felt most powerfully when it obstructed marriage and gendered family capabilities, with men typically expected to bear financial burdens and women tasked with caring and household capabilities. On the other hand, receiving family support and undergoing treatment could mitigate some powerful aspects of leprosy stigma. This study thereby suggests the applicability of WMM in leprosy. However, our findings also suggest that WMM may not fully mitigate societal leprosy-related stigma, as respondents across the full sample highlighted concealment to preserve *ijjat.*

Our findings affirm that stigma is perpetuated by deeply entrenched cultural beliefs of leprosy attributing leprosy to *karma* or *a curse*. These reflect fatalistic beliefs—those viewing health impacts as predetermined and inevitable. This facilitates *apahelana*, indicating a loss of personhood and thus a loss of personal and family *ijjat*. Our study found that this was related to significant social, economic, and (mental) health implications, especially among those with visible signs, deformities, or disabilities due to leprosy. Fatalistic beliefs are also applied to other health issues in Nepal, such as tuberculosis (Engelbrektsson & Subedi, 2016). Paudel and colleagues emphasize the importance of engaging religious leaders alongside health workers to correct such misconceptions (Paudel et al., 2018). They suggest using the concept of *Karma* as presented in the Bhagavad Gita, the holy scripture of Hinduism, which defines *Karma* as the skill of performing one’s actions in the present rather than it being predetermined (Prabhupada, 1972, as cited in Paudel et al., 2018). Future stigma reduction efforts could adopt a similar approach to separate leprosy from prevailing fatalistic understandings.

Personal and family *ijjat* emerged as central cultural construct closely tied to all cultural capabilities that mattered most for both men and women. In Nepalese societies, *ijjat* typically refers to honor and prestige tied to a person or the family (Kohrt & Harper, 2008). Its significance as cultural capital in Nepalese communities is previously described, with *ijjat* defined as social currency that is negotiated, earned, and lost, with physical and personal manifestations through feelings of pride, guilt, and shame, and impacting one’s confidence and well-being (Liechty, 2003). Moreover, *ijjat* is argued to be a gendered construct, referred to as “a complex configuration of interrelated, nuanced understandings, activities, rules and assets, which provides a gendered framework for directing the practices, beliefs and experiences of urban Nepalis” (Homan, 2016, p. x). Our findings support this notion as we observed WMM to differ between men and women, particularly within the family. Further, our findings relating *ijjat* to stigma and health complement those of a prior literature review on mental health stigma and well-being in Nepal (Gurung et al., 2022). Gurung and colleagues used the WMM framework in their analysis of the literature and identified prestige, social acceptance, productivity, marriage, and privacy as key cultural factors influencing mental health stigma. Complementing this work, our study elucidates the cultural capabilities relating to *ijjat* and their interaction with leprosy-related stigma.

### The Family as a Nexus for Stigma Exacerbation and Mitigation through WMM

Our study found that the family serves as a nexus for interactions that can either contribute to leprosy-related stigma (stigma exacerbation) or foster inclusion and social acceptance (stigma mitigation) through facilitating WMM by helping leprosy-affected persons and family to achieve *ijjat*. Previous studies highlight the significance of leprosy within families, reporting that leprosy can negatively affect the mental health and well-being of both individuals affected by leprosy and their family members. For example, a 2013 study from Nepal found that adolescents with leprosy-affected parents scored higher on depression scales and lower on self-esteem and quality of life scales, compared to adolescents whose parents were not affected by leprosy (Yamaguchi et al., 2013). The dual and central role of the family aligns with findings from a study on the demand for and access to mental health services in Nepal, reporting the family as both the primary detector of mental health issues and a critical pathway to appropriate care, while also noting its potential to act as a barrier (Brenman et al., 2014).

Our findings highlight that leprosy is a threat to personal and family *ijjat*, and that stigma is felt most powerfully through the obstruction of gendered family capabilities and marriage, undermining WMM. These findings align with previous findings from the Asian context. For example, a study on tuberculosis from Bangladesh, Nepal, and Pakistan indicated that tuberculosis disrupted gendered roles of a mother, wife, or daughter-in-law, which legitimized rejection of marriage to a woman with tuberculosis (Hatherall et al., 2019). Other studies have emphasized the challenges faced by individuals affected by leprosy in securing marriage (Van Brakel, 2006; van Brakel et al., 2012), as well as reported marital problems and instances of sexual abuse experienced by Nepali women affected by leprosy (van’t Noordende et al., 2016). In our study, marriage prospects appear to be challenged for both men and women affected by leprosy as well as for unaffected family members. Findings further align with literature applying WMM in other contexts, including Botswana, Chile, Indonesia, and Nigeria, suggesting that these gendered expectations may be common categories shaping stigma across various cultural contexts (Ebenso et al., 2019; Mascayano et al., 2015; Misra et al., 2021; Sopamena et al., in preparation; Yang, Poku, et al., 2021). Lastly, societal gossip and the fear of gossip were found to undermine personal and family *ijjat* and stimulate concealment, which resonates with previous findings highlighting gossip as cultural process facilitating stigma (Poku et al., 2023).

On the other hand, we identified family support and undergoing treatment as key pathway to preserve *ijjat* and mitigate some powerful aspects of stigma. While findings are explorative, these suggest that family support to continue to achieve WMM can be a valuable capability to leverage in stigma reduction efforts. This finding is supported by previous literature. Recent studies from Southern and Far-Western Nepal reported that family support could greatly impact the mental well-being of persons affected by leprosy (Pierneef et al., 2022; van Netten et al., 2021). The positive impact of family support in leprosy-related stigma reduction was also reported in studies from other contexts, through stimulating supportive family environments by family counselling, improving family quality of life, and enhancing resilience among affected persons and their family members (Lusli et al., 2016; van’t Noordende, Bakirtzief da Silva Pereira, et al., 2021a, 2021b).

### Significance of Concealment to Maintain Personal and Family Ijjat

People affected by leprosy and their families often concealed the condition when possible. This is consistently described in previous literature from Nepal, including Western Nepal (Adhikari, Kaehler, et al., 2013; Adhikari, Shrestha, et al., 2013; Adikhari et al., 2014; Marahatta et al., 2018; Pierneef et al., 2022; Sermrittirong & Van Brakel, 2014; Singh et al., 2012; van Netten et al., 2021). Hyland (2000) and Heijnders (2004) studied leprosy concealment in Nepal and described that it keeps social integrity intact, referring to one’s position in the community. Similar patterns have been observed in mental illness in Nepal, where concerns about *ijjat* contribute to concealment and avoidance of healthcare (Brenman et al., 2014; Kohrt & Harper, 2008). In this study, the affected person and family appear to act strategically to help the affected person to manage leprosy and achieve WMM while shielding them from societal stigma, particularly any societal stigma which could be reduced by visibility of people with leprosy who function and achieve their life goals. This may inadvertently deprive them of valuable social opportunities, given that concealment is also linked to psychological burdens and can create a discrepancy between the mild symptoms shown by most persons affected and the severe characteristics of leprosy known to the public, which may contribute to delays in seeking treatment (Engelbrektsson & Subedi, 2018; Pachankis, 2007). These findings underscore the necessity for health facilities and providers to support individuals and families in navigating their decision-making regarding (selective) disclosure and ensure confidentiality and privacy.

### Intersectional Stigma

Our findings identify intersectional stigma as ethnicity/caste/geographic location and gender appeared as key factors influencing experiences, leading to distinct forms and outcomes of leprosy-related stigma. First, stigma was considered higher among Pahadi society of the Far-Western region of Nepal in the hilly region, predominantly inhabited by individuals who are considered “higher caste,” while stigma was considered lower among Tharu society, who live mainly in the Terai region. These findings are explorative as they are based on several perceptions of respondents. We did not disaggregate our analysis by ethnicity and/or specific geographic location. However, the findings suggest that ethnic groups, grouped under certain castes and residing in certain regions, may experience comparable levels of overall stigma for different reasons. For example, caste-based discrimination may fuel leprosy-related stigma toward Dalits (those of “lower caste”), while status loss may fuel stigma enacted and perceived by Brahmins (those of “higher caste”) (Nepali et al., 2018). This reflects findings by Adhikari, Shrestha, et al. (2013), reporting highest levels of perceived stigma among the “highest caste” and “lowest caste” in Western Nepal.

Second, our findings suggest that women face differential treatment within the family and barriers to healthcare due to their marginalized social status, which may exacerbate stigma and treatment delays. This aligns with a literature review by Dijkstra and colleagues (2017), reporting that women were disproportionately affected by leprosy in endemic areas worldwide. Another review including studies from Nepal, India, Nigeria, Ethiopia, and Brazil identified four main factors contributing to delayed diagnoses in women: social stigma, low status and economic independence, self-stigma, and gender-insensitive leprosy services (Price, 2017). Several other studies have highlighted the disproportionate impact of leprosy and stigma on Nepalese women (Brouwers et al., 2011; Pierneef et al., 2022; Varkevisser et al., 2009; van’t Noordende et al., 2016). A pilot study in Nepal reported that women received almost three times less family support than men and faced negative family responses, such as divorce or abuse (Floyd-Richard & Gurung, 2000). Our study suggests these disparities in family support persist, emphasizing that improving leprosy-related outcomes requires addressing intersectional stigma, echoing the need for further research on intersectional stigma (Jackson-Best & Edwards, 2018; Rai et al., 2020).

### Study Limitations

We acknowledge several limitations of this study. First, the perspectives and experiences were analyzed at the stakeholder group level, distinguishing between men and women to highlight gendered differences in leprosy-related stigma and WMM. This decision was informed by previous research highlighting that the impacts of leprosy and stigma are gendered (van Dorst et al., 2020; van’t Noordende et al., 2016; Varkevisser et al., 2009). This however implied the analytical assumption that “men” and “women” are homogeneous groups. This may have led to overlooking nuanced differences in experiences within these groups regarding their hierarchical roles within the family and society (e.g., mother-in-law vs. daughter-in-law). Furthermore, our gendered findings seem primarily informed by the traditional division of gender roles and thereby are applicable mostly to heterosexual relationships. Individuals who are part of gender or sexual minorities may face compounded forms of discrimination which this study did not identify.

Other social positions such as ethnicity and age may influence the experiences and outcomes of stigma. Although several respondents suggested ethnicity and caste to influence stigma, our analysis was not stratified as such. The average age of respondent groups was approximately 45 years old and over two-thirds of respondents (79%) were aged between 35 and 65 years, while 19.8% of respondents were aged between 20 and 35 years. Only 7.5% of the sample was unmarried. This may have led to the underrepresentation of perceptions and experiences (including WMM) typical for younger individuals (<35 years) and overrepresentation of those typical for middle age. This may have contributed to the family-oriented findings under WMM, predominantly describing capabilities related to mother- and fatherhood. Moreover, many of the married respondents may have been shielded from the most severe forms of stigma. These experiences may thus be underrepresented in our findings. Future research could stratify its analysis by ethnicity or age or focus on specific groups to allow for a deeper exploration of how WMM may vary across ethnic groups and the lifespan. Future research should consider broader or complementary geographic representation to increase understanding of the cultural and gendered factors shaping leprosy-related stigma in different regions and/or communities in Nepal.

Lastly, we included FGDs to address potential difficulties in articulating WMM in interviews and encourage discussion. Although FGDs carry a risk of socially desirable responses, we mitigated this by stimulating discussions on lived experiences. Notably, we observed that responses were largely consistent across both interviews and FGDs. Recognizing that our primarily deductive analytical approach starting with five deductive codes may have limited the coding from the outset as it prioritizes the predefined framework over other perspectives that arise, we incorporated broader questions about living with leprosy, giving respondents freedom to share their experiences openly.

### Engaging With WMM: Implications for Stigma Reduction Efforts

The family plays a pivotal role in maintaining *ijjat* and acts as a nexus for stigma mitigation and exacerbation. However, the pivotal role of family has not been sufficiently recognized in the existing leprosy stigma–reduction interventions. A recent systematic review of leprosy-related stigma reduction efforts showed that the family was specifically targeted or engaged with in only two of the 17 interventions included (Willis et al., 2024). Future research should prioritize developing and evaluating family-based interventions or incorporate family members as a specific target group within existing interventions, enabling the family to help affected persons achieve WMM.

One way that families can provide support is through facilitating the achievement of WMM for persons affected by leprosy. This can be enabled through family counselling services and the integration of family education sessions alongside patient care (see, for example, Lusli et al., 2016). The RESHAPE program in Nepal provides a good example of family involvement in stigma reduction, in which family members and caregivers are co-facilitators in a mental illness stigma reduction program (Rai et al., 2018). This study found that families played an important role in the meaningful participation of service users in mental health training, research, and services, highlighting ways to engage family members in mental health programs (Rai et al., 2018).

In addition, family-focused information, education, and communication (IEC) interventions could address (cultural) beliefs and misconceptions underlying leprosy-related stigma and encourage supportive behaviors to empower individuals affected by leprosy. While public-based IEC strategies have been previously advocated for in the Nepali context (see Adhikari, Kaehler, et al., 2013; Adhikari, Shrestha, et al., 2013), such strategies have faced criticism for their limited effectiveness in changing negative attitudes and behaviors rooted in fear and cultural beliefs (Cross, 2006; Heijnders & Van Der Meij, 2006). A previous study from Nepal showed promise in reducing stigma by targeting IEC strategies through self-care or self-help groups (Muldoon et al., 2022). In another effort, Cross and colleagues assessed the potential of empowerment and self-care initiatives for individuals affected by leprosy in Southern Nepal as a trigger for stigma reduction through their increased social participation (Cross & Choudhary, 2005; Cross et al., 2017). Such strategies could be extended to engage families of persons affected by leprosy to enable them to facilitate WMM for persons affected by leprosy. Future research should focus on developing and assessing the impact of such targeted IEC interventions.

Lastly, (community) health workers can play key roles in simulating engagement of family members in care and treatment. Given that leprosy is often concealed, future efforts should explore how disclosing a leprosy diagnosis within the family can be supported by (community) health workers, while ensuring confidentiality in facilities, as also suggested by Brenman and colleagues in the context of mental health (2014).

Important to note is that such efforts will not be fully successful if not sensitive to intersecting social forces influencing the lived realities of affected persons and their families (Rai et al., 2020). Our study found that girls and women often face greater barriers to care, limited family support, and negative responses, increasing their risk of poor health outcomes and stigma. Future interventions involving the family should therefore consider gender dynamics and structural factors that exacerbate stigma, particularly for girls and women. Efforts should be complemented by strategies targeting other socioecological levels which are crucial to build capacity for stigma reduction at the level of health facilities, systems, and policy (Rai et al., 2020; Stangl et al., 2019; van Netten et al., 2021). For example, establishing counselling centers at health facilities, community centers, schools, and workplaces, along with increasing the number of skilled female healthcare workers, can help prevent gender-specific delays in treatment and address the intersecting vulnerabilities related to leprosy.

## Conclusion

Our study is the first to explore how local understandings of what it means to be a “good” or “respected” person can intensify or mitigate leprosy-related stigma in Nepal. Our study contributes to the literature by highlighting the necessity and untapped potential of engaging with WMM to inform stigma reduction in Nepal. Our findings demonstrate that family involvement and support can provide a targeted strategy to awareness raising, counselling, treatment access and adherence, empowerment, and social participation, supporting the achievement of WMM and overall stigma reduction. Our findings suggest that future efforts should account for gendered family dynamics and address intersecting inequities in research and stigma reduction. The study advances the literature on stigma and WMM by demonstrating its applicability in uncovering cultural mechanisms that intensify and mitigate leprosy-related stigma. This study highlights how this approach can be utilized in other leprosy-endemic countries to unpack the cultural mechanisms shaping leprosy-related stigma across diverse subgroups and contexts.

## Supplemental Material

Supplemental Material - Cultural Mechanisms of Leprosy-Related Stigma: A Gendered Analysis Using the What Matters Most Framework in Far-Western Nepal Supplemental Material for Cultural Mechanisms of Leprosy-Related Stigma: A Gendered Analysis Using the What Matters Most Framework in Far-Western Nepal by Marlies J. Visser, Eliza A. KC, Yoslien Sopamena, Pirt B. Bist, Sita Bist, Madhusudan Subedi, Nand Lal Banstola, Sarju S. Rai, Lawrence H. Yang, D. Dadun, and Ruth M. H. Peters in Qualitative Health Research

Supplemental Material - Cultural Mechanisms of Leprosy-Related Stigma: A Gendered Analysis Using the What Matters Most Framework in Far-Western Nepal Supplemental Material for Cultural Mechanisms of Leprosy-Related Stigma: A Gendered Analysis Using the What Matters Most Framework in Far-Western Nepal by Marlies J. Visser, Eliza A. KC, Yoslien Sopamena, Pirt B. Bist, Sita Bist, Madhusudan Subedi, Nand Lal Banstola, Sarju S. Rai, Lawrence H. Yang, D. Dadun, and Ruth M. H. Peters in Qualitative Health Research
