# Supplementary material for: Cultural Mechanisms of Leprosy-Related Stigma: A Gendered Analysis Using the What Matters Most Framework in Far-Western Nepal
Source: Qual Health Res. 2025 Mar 27;36(4-5):440–55. doi: 10.1177/10497323251318604 (PMC12982553; doi:10.1177/10497323251318604)
Supplement: Supplemental Material - Cultural Mechanisms of Leprosy-Related Stigma: A Gendered Analysis Using the What Matters Most Framework in Far-Western Nepal [file sj-pdf-1-qhr-10.1177_10497323251318604.pdf]

Supplementary Material 1

Table S1. *Interview Items for Data Collection*

**Draft Interview and Probing Questions**
**1. When do you/does the community consider someone to be a “respected” (possible alternatives: ‘proper, good, decent, complete’) person/woman/man in Sudurpaschim/Nepal?**<br>-- Can you elaborate?<br>-- Why is this the case?<br>-- Is there something specific about [context/country] that shapes this?<br><br>**2. What are the things that a person/woman/man has to do or has to achieve in order to be seen as a “respected” (possible alternatives: “proper, good, decent, complete”) person?**<br>-- Can you elaborate?<br>-- Why is this the case?<br>-- Is there something specific about [context/country] that shapes this?<br><br>**3. How would community members act towards a person if they did not achieve… [insert what participant mentioned in terms of respected personhood]?**<br>-- Can you elaborate?<br>-- Why is this the case?<br>-- Is there something specific about [context/country] that shapes this?<br><br>**4. If the community knows that a person has [health condition], can they still be seen as a “respected” (possible alternatives: “good, proper decent or complete”) person?**<br>-- if No, can you describe to me why they will not be seen as a “respected” (alternative: good, decent, proper, complete) person?<br>-- if Yes, can you describe to me why they will still be seen as a “respected” (alternative: good, decent, proper, complete) person?

5. Would having [health condition] have an impact…
…on a person to find or to maintain an existing relationship with a spouse or romantic partner?
…on a person‘s ability to take care of children?
…on a person’s ability to contribute to household roles or responsibilities (e.g. preparing food, cleaning the house, roles in the extended family, duties to the in-laws families)?
…on a person’s access to education?
…on a person’s ability to find or maintain work or hold a business?
…on the status of the family or the family name?
…on a person’s involvement in community, religious or ethnic-related activities (e.g., invitation and participation in birth ceremony, marriage ceremony, funerals, rituals or festivals)?

-- Can you elaborate?
-- Why is this the case?
