# Supplementary material for: Cultural Mechanisms of Leprosy-Related Stigma: A Gendered Analysis Using the What Matters Most Framework in Far-Western Nepal
Source: Qual Health Res. 2025 Mar 27;36(4-5):440–55. doi: 10.1177/10497323251318604 (PMC12982553; doi:10.1177/10497323251318604)
Supplement: Supplemental Material - Cultural Mechanisms of Leprosy-Related Stigma: A Gendered Analysis Using the What Matters Most Framework in Far-Western Nepal [file sj-pdf-2-qhr-10.1177_10497323251318604.pdf]

Supplementary Material 2

Table S2. *Main Themes, Operational Definitions and Subcodes*

Main themes	Operational definition	Subcodes
**Experiences of stigma**[a]	The public and internalized process of negative stereotyping leading to prejudice and discrimination in contexts of power.	Courtesy stigma<br>Internalized stigma<br>Public stigma<br>Structural stigma
**WMM Manhood: Cultural capabilities that matter most to manhood**[b]	The engagement in core cultural capabilities or activities that determine respected personhood ('manhood') in Sudurpaschim Nepal, encompassing key responsibilities and roles in the family, society, and self.	Achieving WMM:<br>• Good character<br>• Educated<br>• Family responsibility and marriage (including work)<br>• Good health<br>• Faithful in religion<br>• Social participation and reputation<br><br>Consequences of not achieving WMM (i.e., not being a proper, respected, complete man)
**WMM Womanhood: Cultural capabilities that matter most to womanhood**[b]	The engagement in core cultural capabilities or activities that determine respected personhood ('womanhood') in Sudurpaschim Nepal, encompassing key responsibilities and roles in the family, society, and self.	Achieving WMM:<br>• Physical appearance (beauty)<br>• Good character<br>• Educated<br>• Family responsibility and marriage (including motherhood)<br>• Good health<br>• Faithful in religion<br>• Social participation and reputation<br>• Work<br><br>Consequences of not achieving WMM (i.e., not being a proper, respected, complete woman)

**WMM and stigma**[b]	Intensifying stigma: The ways in which leprosy-related stigma threatens the core cultural capabilities that determine respected personhood (‘manhood’ or ‘womanhood’) in Sudurpaschim, Nepal.<br><br>Protecting against stigma: The ways achieving core cultural capabilities can protect against leprosy-related stigma in Sudurpaschim Nepal.	WMM intensifying stigma<br>WMM protecting against stigma
**Stigma intersections**[c]	Intersections of leprosy-related stigma with other inequities and forms of social oppression	Age<br>Gender<br>Caste/Ethnicity<br>Geographic location<br>Socioeconomic status<br>Visibility/severity of leprosy or disability

[a] *Operational definition informed by Link & Phelan (2001)*

[b] *Operational definition informed by Misra et al. (2021) and Yang et al. (2014; 2021b)*

[c] *Operational definition informed by Rai et al. (2020)*
